# Fatigue-Crack Detection and Monitoring through the Scattered-Wave Two-Dimensional Cross-Correlation Imaging Method Using Piezoelectric Transducers

**DOI:** 10.3390/s20113035

**Published:** 2020-05-27

**Authors:** Wenfeng Xiao, Lingyu Yu, Roshan Joseph, Victor Giurgiutiu

**Affiliations:** Department of Mechanical Engineering, University of South Carolina, 300 Main Street, Columbia, SC 29208, USA; wxiao@email.sc.edu (W.X.); rjoseph@email.sc.edu (R.J.); victorg@sc.edu (V.G.)

**Keywords:** piezoelectric transducer, fatigue crack, cross-correlation imaging algorithm, crack inspection, crack growth monitoring, structural health monitoring

## Abstract

Piezoelectric transducers are convenient enablers for generating and receiving Lamb waves for damage detection. Fatigue cracks are one of the most common causes for the failure of metallic structures. Increasing emphasis on the integrity of critical structures creates an urgent need to monitor structures and to detect cracks at an early stage to prevent catastrophic failures. This paper presents a two-dimensional (2D) cross-correlation imaging technique that can not only detect a fatigue crack but can also precisely image the fatigue cracks in metallic structures. The imaging method was based on the cross-correlation algorithm that uses incident waves and the crack-scattered waves of all directions to generate the crack image. Fatigue testing for crack generation was then conducted in both an aluminum plate and a stainless-steel plate. Piezoelectric wafer transducer was used to actuate the interrogating Lamb wave. To obtain the scattered waves as well as the incident waves, a scanning laser Doppler vibrometer was adopted for acquiring time-space multidimensional wavefield, followed with frequency-wavenumber processing. The proof-of-concept study was conducted in an aluminum plate with a hairline fatigue crack. A frequency-wavenumber filtering method was used to obtain the incident wave and the scattered wave wavefields for the cross-correlation imaging. After this, the imaging method was applied to evaluate cracks on a stainless-steel plate generated during fatigue loading tests. The presented imaging method showed successful inspection and quantification results of the crack and its growth.

## 1. Introduction

Fatigue cracks are one of the most common causes for the failure of metallic structures [1]. Fatigue loading on cracked structures makes the crack grow to a critical point, and such damage can develop progressively, leading to a catastrophic failure [2]. Increased emphasis on the integrity of critical structures such as pressure vessels, nuclear reactors, civil infrastructure, and aircraft, creates an urgent need to monitor those structures and to detect damage at an early stage to prevent catastrophic failure [3]. Structure health monitoring (SHM) has gained widespread attention as a means for detecting deterioration and damage to evaluate the safety of the structures [4,5,6,7,8]. Ultrasonic Lamb waves have shown a great potential for fatigue-crack detection and evaluation, due to their good sensitivity for small defects in the structures and the long propagation distance. With such advantages, Lamb wave-based SHM is widely used for thin-walled structures [9,10,11].

A variety of transducers, such as electromagnetic acoustic transducers (EMAT) [12,13], air-coupled transducers [14,15,16], fiber optics [17], piezoelectric wafer transducers [18], and angle beam transducers (ABT) [19] were utilized for guided wave generation. Among these transducers, piezoelectric wafer transducers are convenient enablers for generating and receiving Lamb waves in structures, which are widely used for SHM applications [20,21,22]. Compared to conventional transducers, piezoelectric wafer transducers are low profile, lightweight, low-cost, and unobtrusive to structures. These can be permanently bonded on the host structures in large quantities and achieve real-time monitoring of the structural health status [7]. To actuate Lamb waves, the lead zirconate titanate (PZT) transduces electric energy into elastic energy and couples their in-plane motion with the Lamb waves’ in-plane strain on the material surface. It can excite the desired mode using the frequency tuning method [18]. Recently, scanning laser Doppler vibrometer (SLDV) was widely used for high spatial resolution ultrasonic wavefield measurement, due to its advantage in accurate surface velocity measurement over a spatially dense grid [23]. In recent years, the hybrid PZT–SLDV Lamb wave system was reported as a promising approach for inspecting both metallic and composite structures [24,25,26,27].

Data analysis and damage evaluation techniques are of great importance in establishing any Lamb wave-based damage inspection [28]. In the literature, various approaches were developed for damage detection in structures based on the examinations of wave interactions with damage [29,30,31]. Various imaging algorithms were developed to quantitatively evaluate the defects in the structure, such as wavefield-based imaging [32,33], array-based imaging [34], and wavenumber imaging [35]. Recently, the cross-correlation imaging method using the back-scattered waves and incident waves was adopted for damage imaging. Zhu et al. [28] developed a fast real-time imaging method for multiple damage detection in an aluminum plate, by cross-correlating the back-scattered waves with incident waves in the frequency domain. He and Yuan [36] developed a zero-lag cross-correlation (ZLCC) imaging technique to quantitatively evaluate the damage in composites. In addition, an improved imaging technique was developed, which could provide enhanced imaging for multi-site damage, as compared to the original ZLCC method [37]. However, only the back-scattered waves were used in these cross-correlation imaging algorithms for damage detection.

In this paper, an imaging method based on the cross-correlation principle using the incident waves and scattered waves at discontinuity was proposed for fatigue-crack detection and evaluation, using Lamb waves actuated by PZT transducers. The scattered waves, compared to the back-scattered used in the conventional cross-correlation method, contained wave-damage interactions in all directions and, therefore, provided more accurate imaging. To obtain the scattered waves as well as the incident waves, a scanning laser Doppler vibrometer was adopted for acquiring time-space multidimensional wavefield, followed with frequency-wavenumber processing. For proof-of-concept, the approach was first conducted on a 1-mm thick aluminum 2024-T3 specimen, with a fatigue crack. After this, the imaging method was applied for crack growth monitoring on a 1-mm thick stainless-steel specimen undergoing fatigue loading.

The rest of this paper is organized as follows. Section 2 introduces the proposed cross-correlation imaging method using the incident waves and scattered waves of all directions. Section 3 presents the fatigue test setups to generate the fatigue crack. In Section 4, a hybrid PZT–SLDV system is established, followed by the proof-of-concept study of crack inspection and evaluation. Next, the application of crack growth monitoring and quantification is presented in Section 5. In the end, Section 6 summarizes the findings of the study and outlines the areas envisioned for future work.

## 2. Scattered Wave Cross-Correlation Imaging Method

### 2.1. Cross-Correlation Imaging Technique

In recent years, the cross-correlation imaging method for damage detection has attracted researchers’ interest. Zhu et al. [28] originally investigated the cross-correlation imaging method for multiple damage inspection in aluminum plate, by a permanently mounted linear array of piezoelectric wafer transducers. He and Yuan [36] further conducted a cross-correlation imaging method for damage inspection in composite structures using flexural wave signals. A conventional cross-correlation imaging can be formulated between a source wavefield (Ws) and a receiver wavefield (Wr), either in the time domain (t) or in the frequency domain (f) [38]. The imaging condition was defined based on the concept that if both source waves and the receiver waves were extracted separately, these two wavefields would be kinematically coincident at the discontinuities [28], and the magnitude of the cross-correlation values at the discontinuities would be much larger than that of the remaining locations where it would be small or near zero. 

Most of the reported cross-correlation imaging work used incident waves to cross-correlate with the back-scattered waves for damage detection. It depended highly on the back-scattered wave for damage imaging, which limited its application when the back-scattered wave was not significant. Moreover, when the incident waves interacted with damage or defect, these waves were partially reflected and refracted due to the geometric discontinuity, generating scattered waves, as reported in the literature [39,40,41,42]. In this scenario, the damage acted as a new wave source and introduced scattered waves [26,27,28]. These scattered waves not only propagate back to the source (aka back-scattered waves) but also propagated in all directions. The scattered waves were generated by the damage propagated in all directions and, therefore, these all-direction scattered waves contained more information on the overall dimension of the damage, than the back-scattered waves.

### 2.2. 2D Cross-Correlation Imaging Using Scattered Waves

With the advantages of the scattered waves, a 2D cross-correlation imaging method that uses scattered waves of all directions as well as incident waves in the time domain, is proposed for precise damage imaging. The imaging algorithm is expressed as

(1) $$I(x,y)=\int_{0}^{T}v_{incident}(x,y,t)v_{scattered}(x,y,t)dt$$

where $v_{incident}(x,y,t)$ is the incident wave wavefield and $v_{scattered}(x,y,t)$ is the scattered wave wavefield. In this study, wavefields are the out-of-plane velocity of the Lamb wave, with respect to the propagation time *t* and location (*x*, *y*) measured in the plate. I(x,y) is the cross-correlation value at the point (*x*, *y*). With the cross-correlation of all data points (*x*, *y*) calculated, the damage imaging result could then be generated by plotting the cross-correlation values of all data points.

To implement the imaging method, the essential step was to extract the incident waves and the scattered waves successfully. The frequency wavenumber filtering technique was reported and shown as a promising method to separate and extract various wave modes, as well as scattered waves for damage detection [24,25,26,27,28,36,42,43]. It was therefore adopted in this work to extract the desired incident and scattered wavefields. The wavefield in the time-space domain could be converted to the frequency-wavenumber domain, through a three-dimensional Fourier transform (3DFT) method:

(2) $$V(f,k_{x},k_{y})=\int_{-\infty }^{+\infty }\int_{-\infty }^{+\infty }\int_{-\infty }^{+\infty }v(x,y,t)e^{j(2\pi ft-k_{x}x-k_{y}y)}dxdydt$$

where the v(x,y,t) is the Lamb wave wavefield in terms of time variable *t*, and spatial variables *x* and *y*. $V(f,k_{x},k_{y})$ is the converted frequency wavenumber spectrums in terms of frequency *f* and wavenumbers *k_x_* and *k_y_* for *x* and *y*. Next, the incident waves and scattered waves filtering process in the wavenumber spectrum was expressed as the product between the frequency wavenumber spectrum $V(f,k_{x},k_{y})$ and the filter function $F(f,k_{x},k_{y})$, as described in [24]:

(3) $$V_{F}(f,k_{x},k_{y})=V(f,k_{x},k_{y})F(f,k_{x},k_{y})$$

where $V_{F}(f,k_{x},k_{y})$ is the filtered frequency-wavenumber component. Then, the inverse 3DFT method could be used to convert the filtered spectrum back to the time-space wavefield v(x,y,t). Details of the frequency-wavenumber filtering process were reported and can be referred to in [24].

## 3. Fatigue Tests for Crack Generation in Metallic Specimens

**Figure 1:**
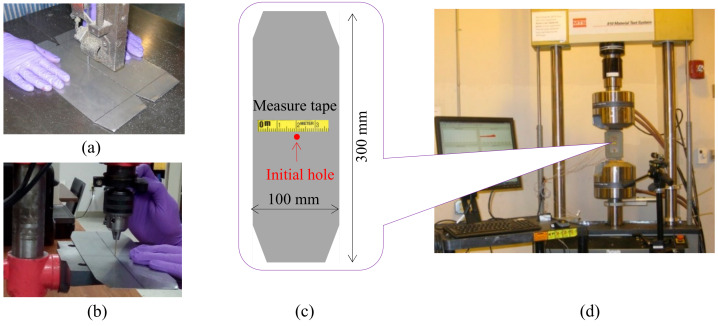
Experimental setup for fatigue testing: (**a**) Specimen preparation; (**b**) drill 1-mm hole at the center of the plate for crack initiation; (**c**) schematic of the fatigue specimen; and (**d**) the overall setup of the fatigue test.

To prepare the cracked specimens, fatigue tests on the selected metallic materials were conducted using the material test system (MTS). Specimens of 100 mm width and 300 mm length were cut using a vertical band saw, as demonstrated in Figure 1a. The geometry was selected such that it was sufficiently wide to allow a long crack to form, and there was enough space for the Lamb wave to propagate and interact with the crack. The top and bottom boundaries were trimmed to fit with the grips of the MTS machine, as shown in Figure 1c. A 1-mm diameter hole was created by drilling at the center of the specimen to initiate crack growth during fatigue loading (Figure 1b). The overall setup for fatigue testing is given in Figure 1d. 

The fatigue loading was set at a tensile–tensile format to make sure the specimen did not buckle. During fatigue testing, the load level applied to the specimen was crucial for successful cracking. In this test, the load level was selected based on the yield limit of the specimen, which depend both on the material properties and geometry of the specimen. The highest load level was set at 65% of the yield strength, and the lowest level was set at 6.5%. The frequency of the applied cyclic fatigue loading was 4 Hz. A 1-mm aluminum 2024-T3 plate and a 1-mm stainless steel plate were used. A small piece of the self-adhesive measure tape was placed on the specimen surface to measure the rough crack length. When the desired crack length was observed, the fatigue test was then paused, and the specimen was removed to perform the ultrasonic Lamb wave inspection and evaluation, as described in the following sections.

## 4. Crack Imaging Inspection and Evaluation Using Piezoelectric Transducers

### 4.1. PZT Lamb Wave Actuation

**Figure 2:**
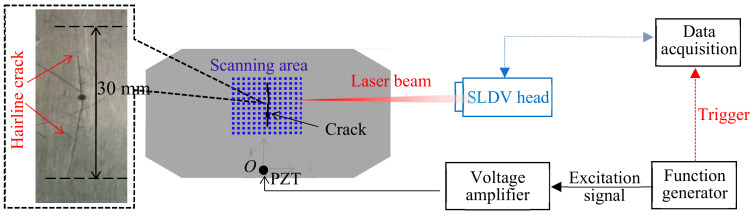
The schematic setup of the lead zirconate titanate (PZT) Lamb wave inspection system for crack detection combined with SLDV to obtain the multi-dimensional scattered waves as well as the incident waves.

In this section, a 1-mm aluminum specimen with a 30-mm fatigue hairline crack was used for the proof-of-concept study. A hybrid system was configured by using the lightweight PZT (Steminc SM412, 0.5 mm thick and 7 mm diameter) for the Lamb wave actuation. It was reported that the Lamb wave frequency would affect the interaction between the Lamb wave and the damage, thus affecting its sensitivity to damage detection [44]. For Lamb wave actuation by PZT in the 1-mm aluminum 2024-T3 plate (Mcmaster-carr, Elmhurst, IL, USA), a single A_0_ mode excitation could be achieved at 120 kHz, due to the tuning effect [39]. Therefore, the excitation frequency of 120 kHz was selected for the single A_0_ mode inspection of the fatigue crack. The overall experimental setup is given in Figure 1. A function generator (AFG 3022C, Tektronix, Beaverton, OR, USA) was used to generate a 3-count tone-burst signal at the central frequency of 120 kHz. This excitation signal was amplified to 140 Vpp by the power amplifier (HSA4014, NF, Yokohama, Japan) and applied to the PZT transducer. At the same time, a trigger signal from the function generator was used to synchronize the data acquisition of the SLDV. On the sensing side, the SLDV (PSV-400-M2, Polytec, Waldbronn, Germany) system was used for acquiring a time-space multidimensional wavefield with a high spatial resolution [25]. The laser beam was placed normal to the plate surface such that only the out-of-plane velocities were measured. The cartesian coordinates (*x*, *y*) were defined with the PZT center as the coordinate origin *O*, as shown in Figure 2. The SLDV conducted an area scan over a predefined 2D scanning grid with a 0.5 mm spatial resolution. To improve the signal quality, the measurement at each scan point was averaged 100 times. To further improve the measurement quality, the well-acknowledged reflective tape was used to enhance the surface reflection [25,26,27].

**Figure 3:**
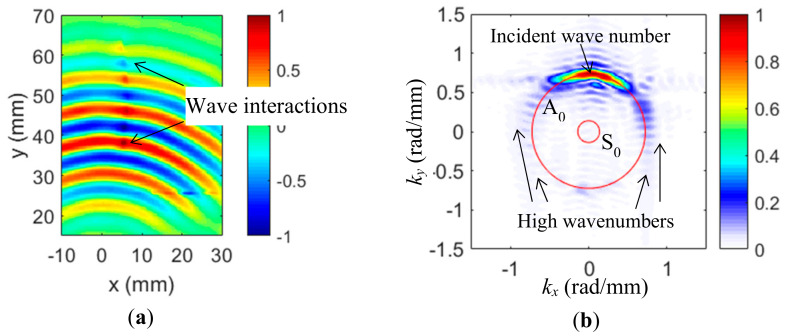
Experimental wavefield and wavenumber spectrum: (**a**) 2D wavefield at 35 µs showing the wave interactions at the crack; (**b**) wavenumber spectrum at 120 kHz using the 3DFT method.

The Lamb wavefield *v*(*x*, *y, t*) at selected time *t* = 35 µs is given in Figure 3a. All wavefields in the study were individually normalized by their own maximum amplitude to show the wave propagation or interactions at the damage. It could be seen that the Lamb waves were successfully captured with the wave propagation pattern, along the positive *y*-axis demonstrated. Additionally, at the crack location, wave-crack interactions with stronger wave intensity at the crack location were observed, as compared to the wave intensity of the pristine location. The time-space wave-crack interaction wavefield was transferred to the frequency-wavenumber domain by the 3DFT method. At the excitation frequency of 120 kHz, the 2D wavenumber spectrum is shown in Figure 2b and the theoretical wavenumber spectrum was also plotted for comparison. It was found that the dominant A_0_ mode was achieved with the PZT actuated at 120 kHz, which was consistent with the theoretical prediction in [39]. The wavenumber component of the A_0_ mode was dominant in positive *k_y_* because the propagation direction of the incident waves was along the positive y-axis direction. Additionally, some weak high wavenumber components larger than the A_0_ mode were observed in all directions.

### 4.2. Crack Imaging and Evaluation

**Figure 4:**
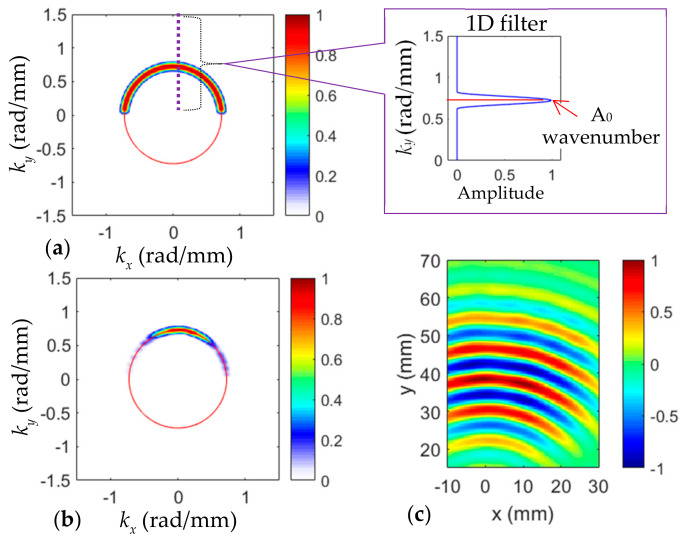
Wavenumber filtering process for the extraction of incident waves: (**a**) Designed 2D band-pass filter at 120 kHz for incident wave filtering; (**b**) filtered incident waves spectrum; and (**c**) the extrapolated incident waves wavefield at 35 μs using the inverse 3DFT method.

The cross-correlation imaging technique using the incident waves and crack-induced scattered waves of all directions was implemented to quantify and evaluate the crack. For the incident wave filtering, a bandpass filter was designed to retain only the incident waves of positive *k_y_*. The wavenumber filter of 120 kHz is given in Figure 4a. The red line is the theoretical A_0_ wavenumber. The bandpass filter details are illustrated in the 1D filter (*k_y_* = 0 to 1.5 rad/mm) with the theoretical A_0_ value plotted. By applying the filter to the experimental Lamb wave wavenumber spectrum, the wavenumbers of incident waves are filtered out. The filtered incident wave spectrum of 120 kHz is given in Figure 4b. By comparing to the original spectrum (Figure 3b), it can be noted that only the incident A_0_ was retained. The time-space wavefield of incident waves was then obtained by the inverse 3DFT. At 35 µs, the filtered incident wavefield is shown in Figure 4c. Only the incident waves were retained, compared to the original wavefield.

**Figure 5:**
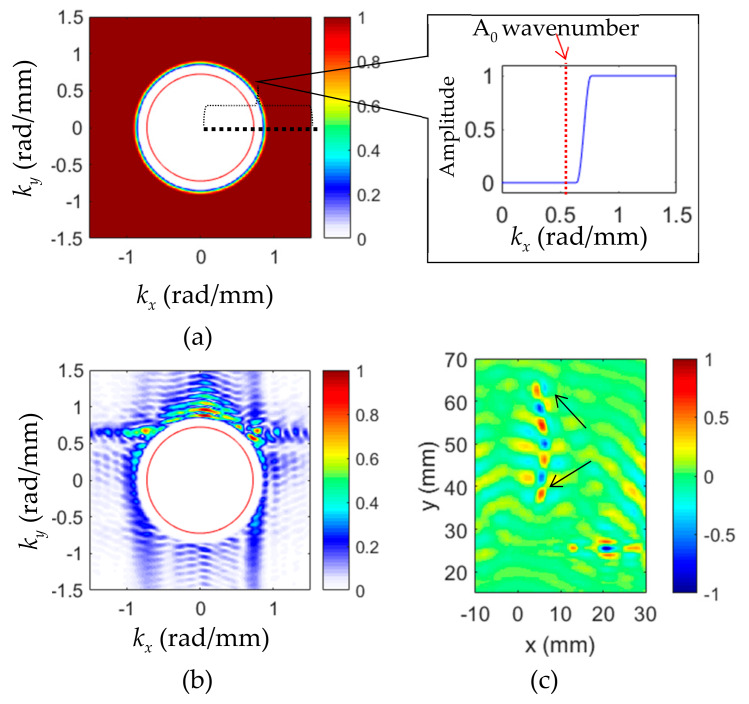
Wavenumber filtering process for the extraction of scattered waves in all directions. (**a**) Designed high-pass filter at 120 kHz for scattered wave filtering; (**b**) filtered scattered wave spectrum; and (**c**) extrapolated scattered wavefield by inverse 3DFT method.

Lamb wave propagation in terms of frequency-wavenumber characterization in a structure depends on the frequency and plate thickness [30]. For waves propagating in a plate, when a crack happens, these waves would be modified in many ways by the crack characteristics including size, depth, shape, and orientation. When cracking happens, the thickness at the crack changes, and this thickness change might introduce scattered waves that propagate with new wavenumber waves in all directions. In Figure 2b, it can be seen that other than the dominant A_0_ mode, incident wavenumber, weak higher wavenumber components were observed in all directions. Therefore, it was assumed that these high wavenumber components were related to the scattered waves generated at the crack. To obtain the scattered waves, a high-pass filter of all directions was designed. At 120 kHz, the wavenumber filter is illustrated in Figure 5a with the 1D filter (*k_x_* = 0 to 1.5 rad/mm) showing the details. By applying the high-pass filter, the scattered-wave wavenumbers were filtered out. The filtered spectrum at 120 kHz with the theoretical A_0_ wavenumber is given in Figure 5b. It was found that only higher wavenumbers were retained. The scattered wave wavefield was then obtained by inverse 3DFT. At 35 µs, the filtered scattered wavefield is given in Figure 5c. Compared to Figure 3a, only the scattered waves induced at the crack were retained. The crack location and dimension could be roughly estimated from the scattered wave wavefield.

**Figure 6:**
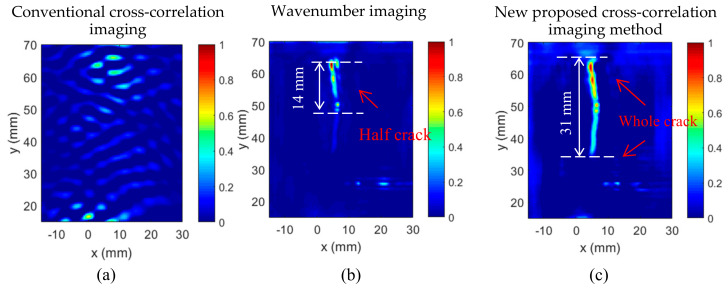
Imaging method comparison: (**a**) Original back-scattered cross-correlation imaging; (**b**) conventional wavenumber filtering imaging result of the hairline crack showing only half crack; and (**c**) the proposed cross-correlation imaging result presenting a more accurate image of the entire crack.

To analyze and evaluate the proposed cross-correlation imaging result. First, conventional cross-correlation imaging using back-scattered waves cross-correlated with the incident waves was obtained, as given in Figure 6a. The result showed that crack was not imaged using the conventional cross-correlation imaging method. This was because the conventional cross-correlation imaging method depend on the back-scattered waves, to a great extent. However, in this research, the hairline crack was aligned with the wave propagation direction, thus, barely back-scattered waves were generated by the crack, failing the crack imaging. Next, the crack imaging using the wavenumber imaging method was obtained, as shown in Figure 5b, which was generated directly from the scattered waves. Details of the wavenumber filtering imaging algorithm can be found in [24]. Figure 6b shows that only half of the crack was imaged by the wavenumber filtering method, and the crack length was estimated to be around 14 mm, with a 53.3% error.

Finally, the proposed cross-correlation algorithm in the Equation (1) was applied using the filtered incident waves and scattered waves to image the crack. The crack imaging result is shown in Figure 6c. It could be seen that the entire crack was imaged successfully, and the crack dimension matched well with the actual crack. Additionally, the initial hole at the center of the crack was also demonstrated in the imaging result. Moreover, the crack was estimated at 31 mm, with a 3.3% error. Compared with the wavenumber imaging result, the proposed cross-correlation algorithm significantly improved the imaging of the hairline crack. The imaging method was based on the wave intensity, but the cross-correlation algorithm measured the similarity of the incident waves and the scattered waves of all directions. Therefore, even the scattered wave intensity of the bottom part was not as strong as the upper part, it could still be imaged clearly.

## 5. Crack Growth Monitoring and Evaluation

**Figure 7:**
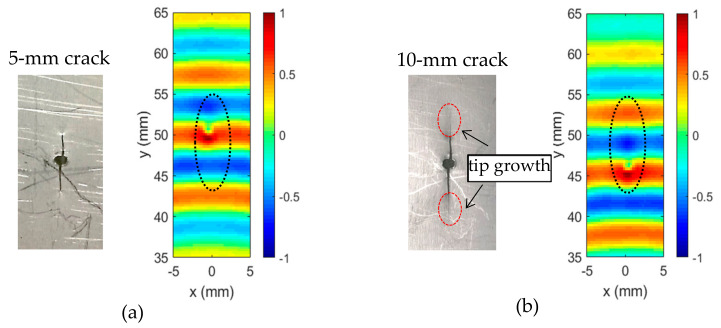
Wavefield comparison at 35 µs showing wave interactions with cracks of different lengths: (**a**) 5-mm fatigue crack and (**b**) 10-mm fatigue crack.

The cross-correlation imaging method was successfully applied for fatigue crack imaging and evaluation. In this section, the crack growth monitoring in a 1-mm stainless-steel specimen was conducted. To monitor the crack growth, fatigue loading was paused at two selected crack lengths (5 mm and 10 mm), and the specimen was removed from the MTS machine to perform the ultrasonic Lamb wave inspection and evaluation. Note that the 5 mm crack was opened with an initial hole at the specimen center in this steel specimen, as shown in Figure 7a. However, the hairline crack growths at the crack tips were observed for 10-mm cracks, as shown in Figure 7b.

The same ultrasonic Lamb wave inspection setups were used for the crack growth inspection. The time–space wavefields of 5-mm crack and 10-mm crack were acquired through the PZT-SLDV system. At 35 µs, the wavefields of the two cracks are given in Figure 7. Very weak wave-crack interactions were observed in the wavefields. Compared to the 5-mm crack case, a stronger wave-crack interaction was noted for the 10-mm crack case.

**Figure 8:**
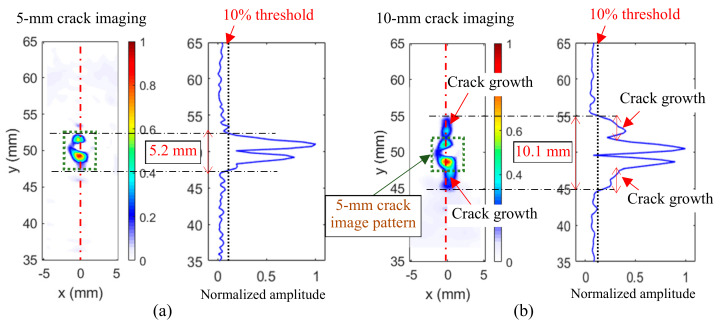
The cross-correlation imaging results: (**a**) 5-mm fatigue crack and (**b**) 10-mm fatigue crack.

The proposed cross-correlation imaging method was applied to the incident wave and the scattered wave wavefields filtered out by the wavenumber filtering method for both the 5-mm crack and the 10-mm crack. Both cracks were successfully imaged, as shown in Figure 8a and b, respectively.

The 5-mm crack imaging result (Figure 8a) showed that the crack was successfully imaged with the open initial hole at the center. To quantify the crack, the normalized cross-correlation values from *y* = 35 mm to *y* = 65 mm at *x* = 0 (as indicated in Figure 8a with red dotted line) was further plotted; as shown in Figure 8a. At crack-free locations, the cross-correlation values were nearly zero; while at the crack locations, the cross-correlation values were significantly larger. It straightforwardly demonstrated that the cross-correlation values at the crack location were much larger than that of the crack-free location, including the initial hole location. In this study, a threshold was set to 10% (the black dotted line in Figure 8a), and this value was selected based on the noise level of the result. The crack length was estimated at 5.2 mm with an 4% error, compared to the actual crack length.

Figure 8b shows the 10-mm crack was successfully imaged. Compared to the 5-mm crack image, it was observed that, both, the patterns of the initial hole and the 5-mm crack were observed in the image. Additionally, crack growth patterns were noted at the two crack tips. Similarly, the normalized cross-correlation values from *y* = 35 mm to *y* = 65 mm at *x* = 0 (as indicated in Figure 8b with red dotted line) were further plotted (Figure 7b) to quantify the crack. Compared to the result in Figure 8a, the increased cross-correlation values of the crack growth were observed. To be consistent with the 5-mm crack case, the same threshold of 10% (the black dotted line in Figure 8b) was used to quantify the crack length. The 10-mm crack length was estimated at 10.1 mm, with a 1% error, compared to the actual crack length. Moreover, smaller cross-correlation values were observed at the crack growth locations than the 5-mm crack locations, which might be due to the narrower hairline crack growth, as compared to the initial open crack. Based on the estimated crack lengths, the crack growth was determined to be 4.9 mm with a 2% error, compared to the actual 5-mm crack growth. The crack growth quantification results are given in Table 1. The results of the crack growth imaging showed that the proposed imaging algorithm was robust, to accurately quantify the cracks and monitor the crack growth.

## 6. Conclusions

This paper presented a 2D cross-correlation imaging method for fatigue crack inspection and monitoring, using a hybrid PZT–SLDV Lamb wave system. First, a cross-correlation imaging method using the incident waves and scattered waves of all directions was introduced. The frequency-wavenumber filtering method was utilized to extract the incident waves and the scattered waves. Then, the fatigue test was conducted in an aluminum specimen and a stainless-steel specimen for the crack generation. Next, the proof-of-concept study was conducted in the aluminum specimen with a 30-mm hairline crack. The crack was successfully detected and evaluated, and it was precisely quantified with only a 3.3% error. Finally, the fatigue crack growth monitoring was carried out in the stainless-steel plate, under fatigue loading. The fatigue cracks at two selected lengths, 5 mm and 10 mm, with 5-mm hairline crack growth, were investigated. The 5-mm open crack was estimated at 5.2 mm, with a 4% error and a 10-mm crack was estimated at 10.1 mm with a 1% error. The 5-mm crack growth quantification showed that the crack growth was quantified with as low as a 2% error.

This study was focused on the development of the cross-correlation imaging method, using incident waves and scattered waves of all directions, and the demonstration of its application for fatigue caused crack detection and quantification. The use of the scattered waves of all directions had the advantage of containing more information about the fatigue crack regarding the overall dimension of the crack. The frequency-wavenumber filtering method could successfully extract the incident waves and scattered waves of all directions. The cross-correlation imaging method precisely imaged the hairline crack, providing the location, dimension, and shape. It significantly improved the imaging of the fatigue crack, as compared to the conventional wavenumber filtering imaging method. The application of the proposed imaging method for fatigue crack growth monitoring demonstrated that it could successfully monitor the fatigue crack growth.

The imaging method presented here relied on the correlation between the incident waves and the scattered waves induced by the defect, as well as the successful acquisition of these waves. It is believed the defect had a great chance to be detected, regardless of the structural material or the defect shape, as long as there were sufficient scattered waves caused by the defects and they could be extracted. The future work would, therefore, be focused on extending its application to different structural materials, such as composites, and to more complex defects with highly irregular profiles.

The presented cross-correlation imaging method using Lamb waves for defect detection was affected by various parameters pertinent to the waves and the defect itself, as well. Lamb wave frequency was one of the main factors, since the related wavelength affected the wave interaction and scattering at the defect. The scattering was also significantly affected by the properties of the defect, including its dimension and profile. It could be noted that the cracks in the aluminum and the stainless-steel plate were different, due to the different deformation and fracture behaviors of these materials. The two cracks, therefore, caused different scatterings in the two plates and subsequently different imaging results in the applications. Additionally, the loading conditions, such as the load level and the cyclic setup would also affect the formation and characteristics of the cracks. A systematic study on these factors that might eventually affect the imaging results is highly recommended for future studies, to further improve the imaging quality.

## Figures and Tables

**Table 1:** The crack and crack growth quantification results.

Actual Crack Length	Estimated Crack Length	Error	Estimated Crack Growth	Error
5 mm	5.2 mm	4%	4.9 mm	2%
10 mm	10.1 mm	1%		
